# Association between hyperuricemia and atrial fibrillation in rural China: a cross-sectional study

**DOI:** 10.1186/s12872-015-0089-y

**Published:** 2015-09-01

**Authors:** Guo-Zhe Sun, Liang Guo, Jun Wang, Ning Ye, Xun-Zhang Wang, Ying-Xian Sun

**Affiliations:** Department of Cardiovascular Medicine, The First Hospital of China Medical University, 155 Nanjing Street, Heping, Shenyang, Liaoning 110001 China; Heart Institute, Cedars Sinai Medical Center, Los Angeles, 90048 CA USA

## Abstract

**Background:**

To explore the association between atrial fibrillation (AF) and serum uric acid (SUA) in a general population in rural China.

**Methods:**

From January 2013 to August 2013, we performed a cross-sectional study involving 11,956 permanent residents ≥ 35 years old in the rural Liaoning province of China. All participants completed a questionnaire, had a physical examination, and underwent an electrocardiogram (ECG) and echocardiogram. AF was diagnosed from ECG findings and/or a history of physician-confirmed AF. Blood samples were drawn for laboratory analyses and hyperuricemia was defined as an SUA level > 7.0 mg/dL in men and > 5.7 mg/dL in women, based on the NHANES-III laboratory definition. Logistic regression analyses were performed to estimate the crude and independent associations between hyperuricemia and the prevalence of AF.

**Results:**

A total of 139 participants were diagnosed with AF, of which, 72 were self-reported, 45 were ECG-diagnosed, and 22 were both. There was a higher prevalence of AF in participants with hyperuricemia than those with normal SUA levels (2.4 vs. 1.0 %; *P* < 0.001). The odds ratios (OR) and 95 % confidence intervals (CI) were 2.37 (1.61–3.49) when compared to participants with normal SUA. After adjustment for other cardiovascular and AF risk factors, the independent association remained (OR = 1.94, 95 % CI: 1.26–3.00). Similar associations were observed between SUA as a continuous variable and AF prevalence (adjusted OR = 1.20, 95 % CI: 1.06–1.36). The independent associations were significant in men (*Ps* < 0.05) but not in women (*Ps* > 0.05), although the interaction logistic regression analyses presented these differences as not being statistically significant (*Ps* > 0.05).

**Conclusions:**

SUA is positively associated with the prevalence of AF in rural China.

## Background

Atrial fibrillation (AF) is one of the most common cardiac arrhythmias and is associated with overall mortality and mortality from cardiovascular disease [1, 2]. Advancing age, male gender, hypertension, diabetes mellitus, obesity, heart failure, myocardial infarction (MI), and alcohol consumption are the major risk factors for the development of AF [3–6]. The prevalence of AF is expected to increase dramatically over the next few decades as the general population ages and improved cardiovascular therapies keep people with cardiovascular disease alive longer [7]. Identifying all the risk factors for AF will help to create population-based strategies to deal with this serious health problem.

Serum uric acid (SUA) is a risk factor for cerebrovascular and coronary artery disease, as well as for hypertension, metabolic syndrome, and kidney disease [8], though only a few cross-sectional studies in Japan [9], Turkey [10], and China [11–13] have reported a positive association between hyperuricemia and the prevalence of AF. Also, population-based prospective cohorts showed that hyperuricemia was associated with a high risk of AF [14, 15]. However, all of these cross-sectional studies enrolled hospital patients only rather than individuals from the general population. Furthermore, only the study in Japan analyzed the effect of gender on the association between hyperuricemia and AF, reporting that the independent association was observed in women only. Therefore, the current study was designed to explore the association between SUA and AF in a general population from rural China. The study also analyzed the effect of gender on the independent association.

## Methods

### Study population

A representative sample of men and women ≥ 35 years of age from rural areas of Liaoning Province were recruited between January 2013 and August 2013 using a multi-stage, randomly stratified, cluster-sampling scheme. In particular, three counties (Dawa, Zhangwu, and Liaoyang) were randomly selected from Liaoning Province. One township near a city in each county was randomly selected giving a total of three townships. Six to eight villages from each township were randomly selected to give a total of 26 rural villages. All of the eligible permanent residents aged ≥ 35 years from each village (*n* = 14,016) were invited to participate in the study, and 11,956 (85.3 %) agreed to do so; women who were pregnant, or people who had cancer or any mental disorders were excluded from the study.

The study was carried out with pre-approval granted by the Ethics Committee of China Medical University (Shenyang, China). Written consent was obtained from all participants after they had been informed of the study’s objectives, benefits, medical procedures, and confidentiality safeguards for personal information. In the case of an illiterate participant, written informed consent was obtained from the appropriate legal proxy.

### Data collection and measurement

Data were collected during a single clinic visit by cardiologists and trained nurses using a standard questionnaire in a face-to-face interview. All potential investigators had received training on the objectives of the study, how to administer the questionnaire, the standard methods of measurement, the importance of standardization, and study procedures. Only those who earned a perfect score on a post-training test were allowed to participate as study investigators. During data collection, the inspectors received further instructions and support.

Data on demographic characteristics and medical history of AF, MI, hypertension, diabetes mellitus, lifestyle risk factors, and family history of AF were obtained, as described above, by interview with the standardized questionnaire. There was a central steering committee with a subcommittee for quality control that made sure all data were collected according to well-known standards.

According to the American Heart Association, blood pressure (BP) was measured three times at two-minute intervals after at least five minutes of rest using a standardized automatic electronic sphygmomanometer (HEM-907; Omron, Kyoto, Japan). Two doctors checked the calibration of the Omron device every month using a standard mercury sphygmomanometer according to the British Hypertension Society protocol [16]. The participants were advised to avoid caffeinated beverages and to exercise for ≥ 30 min before the measurement. During the measurement, the participants were seated with their arms supported at the level of their hearts. The mean of three BP measurements was calculated and used in all analyses.

Weight and height were measured to the nearest 0.1 kg and 0.1 cm, respectively, with the participants in lightweight clothing without shoes. The body mass index (BMI) was calculated as weight in kilograms divided by the square of the height in meters. Waist circumference (WC) was measured at the umbilicus to the nearest 0.1 cm while the participants were standing following a normal expiration.

Fasting blood samples were collected in the morning after ≥ 8 h of fasting for all participants. Blood samples were obtained from an antecubital vein using BD Vacutainer tubes containing EDTA (Becton, Dickinson and Co., Franklin Lakes, NJ, USA). Serum was subsequently isolated from whole blood, and all serum samples were frozen at −20 °C for testing at a central, certified laboratory. Fasting blood glucose (FBG), total cholesterol (TC), triglycerides (TG), SUA, and other routine blood biochemical indices were analyzed enzymatically on an auto-Analyzer (Olympus AU640; Olympus, Kobe, Japan, or Bayer RA-XT; Bayer Diagnostics, Tarrytown, NY, USA) using kits (Bayer Diagnostics). The laboratory measurements were calibrated and verified following analysis of biochemical indices and the results met the national standards of measurement (CNAS certificate of accreditation No.L0467, quality index *U* = 0.006 (k = 2)).

Twelve-lead resting, ten-second electrocardiograms (ECGs) were performed on all participants by well-trained cardiologists using an electrocardiography machine (MAC 5500; GE Healthcare, Little Chalfont, Buckinghamshire, UK). The results were analyzed automatically by the MUSE Cardiology Information System (version 7.0.0; GE Healthcare). ECG-based diagnoses of AF were confirmed by at least two independent cardiologists.

Echocardiograms were obtained using a commercially available Doppler echocardiograph (Vivid; GE Healthcare) with a 3.0-MHz transducer. The transthoracic echocardiogram included M-mode, two-dimensional, spectral and color Doppler with subjects in the supine position. Echocardiogram analyses and readings were performed by three doctors specialized in echocardiography, and two other specialists were called in if questions or uncertainty arose. Measurements were performed according to the recommendations of the American Society of Echocardiography. M-mode images were used to measure and calculate the left ventricular ejection fraction (LVEF) [17].

### Definitions

AF was diagnosed based on a previous history of AF (previously diagnosed by a physician) and/or evidence of AF on the ECG (absence of consistent P waves, presence of rapid irregular f waves with a frequency of 350–600 beats per minute, and an irregular ventricular response). Left ventricular hypertrophy diagnosed by ECG (ECG-LVH) was identified using the Cornell Criteria expressed as voltage and QRS duration product: (RaVL + SV3) × QRS duration > 2,440 mm*ms in men and (RaVL + SV3 + 8 mm) × QRS duration > 2,440 mm*ms in women [18]. Left ventricular systolic dysfunction was defined as an LVEF < 0.5 based on M-mode echocardiography. Hyperuricemia was defined as an SUA level > 7.0 mg/dL in men and > 5.7 mg/dL in women, based on the NHANES-III laboratory definitions [19].

### Statistical analysis

All statistical analyses were performed using SPSS 17.0 software (SPSS Inc., Chicago, IL, USA). Differences between groups were compared using a two-tailed Student’s *t*-test for continuous variables and a χ^2^ test for categorical variables. The age- and gender-specific prevalences of AF among participants with both normal SUA levels and hyperuricemia were calculated, and univariate and multivariate logistic regression analyses were performed to estimate the crude and independent association between SUA and the presence of AF. Interaction regression models were used to test the difference in the association of SUA with AF prevalence between men and women. Data are expressed as odds ratio (OR) and 95 % confidence interval (CI), mean ± standard deviation, or frequency and percentage; a *P* < 0.05 was considered as statistically significant.

## Results

### Characteristics of the study population

Of the original 11,956 participants, 618 had incomplete data and were excluded from the analysis, leaving a total of 11,338 participants (5,170 men and 6,168 women) with a mean age of 53.8 years. The subjects with hyperuricemia were older than those with normal SUA levels (*P* < 0.001), and there was a higher percentage of men than women in this group (*P* < 0.001) (Table 1). Participants with hyperuricemia had significantly higher WCs, BMIs, systolic and diastolic BPs, FBG, TC, and TG levels (all *P*s < 0.001). The hyperuricemia group also had a higher percentage of alcohol drinkers and had a higher prevalence of MI, and ECG-LVH than the normal SUA level group (all *P*s < 0.001). The prevalence of AF was significantly higher in participants with hyperuricemia than those with normal SUA (*P* < 0.001).

**Table 1:** Characteristics of the study population

Variable	Normal SUA level	Hyperuricemia	*P*
	(*n* = 9,909)	(*n* = 1,429)	
Age, y	53.7 ± 10.5	54.9 ± 10.9	<0.001
Sex, male	4396 (44.4)	774 (54.2)	<0.001
BMI, kg/m^2^	24.6 ± 3.6	26.4 ± 3.9	<0.001
WC, cm	81.6 ± 9.6	87.9 ± 9.9	<0.001
SBP, mmHg	141.2 ± 23.4	145.9 ± 23.8	<0.001
DBP, mmHg	81.5 ± 11.5	85.7 ± 12.7	<0.001
FBG, mmol/L	5.88 ± 1.65	6.05 ± 1.47	<0.001
TC, mmol/L	5.18 ± 1.06	5.58 ± 1.23	<0.001
TG, mmol/L	1.53 ± 1.30	2.41 ± 2.34	<0.001
Current smoker	3478 (35.1)	502 (35.1)	0.982
Current drinker	2107 (21.3)	418 (29.3)	<0.001
History of MI	101 (1.0)	30 (2.1)	<0.001
LVEF < 0.5	1126 (11.8)	144 (10.4)	0.131
ECG-LVH	799 (8.1)	215 (15.0)	<0.001
AF	104 (1.0)	35 (2.4)	<0.001
Family history of AF	306 (3.1)	38 (2.7)	0.377

Note: data are expressed as mean ± standard deviation or *n* (%)

*AF* atrial fibrillation, *BMI* body mass index, *DBP* diastolic blood pressure, *ECG-LVH* left ventricular hypertrophy detected by electrocardiography, *FBG* fasting blood glucose, *LVEF* left ventricular ejection fraction, *MI* myocardial infarction, *SBP* systolic blood pressure, *SUA* serum uric acid, *TC* total cholesterol, *TG* triglycerides, *WC* waist circumference

### Prevalence of AF by SUA level

**Fig. 1:**
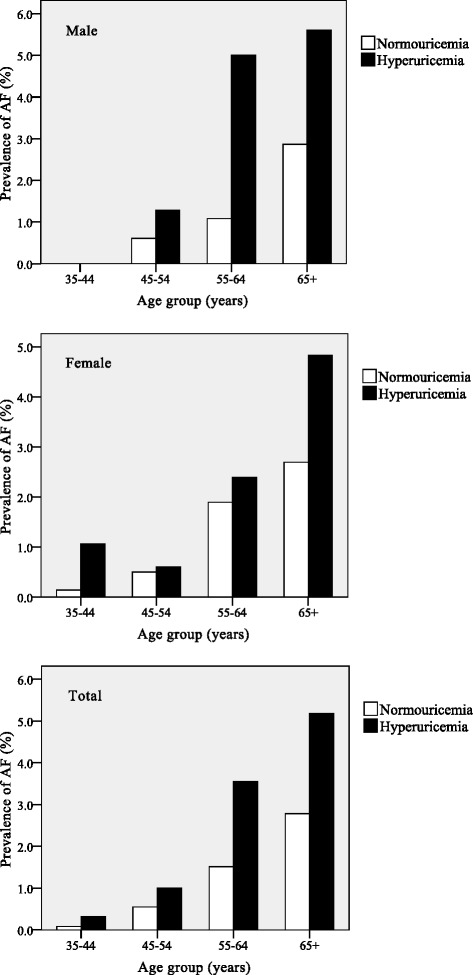
Age-specific prevalence of atrial fibrillation by serum uric acid levels. The prevalence of AF in men (*top*), women (*center*), and the total population (*bottom*)

There were 139 participants with AF, and the age-specific prevalence of AF by SUA level is summarized in Fig. 1. Among these AF cases, 72 were self-reported, 45 were ECG-diagnosed, and 22 were both. The prevalence of AF rose steeply with advancing age and was higher in the group with hyperuricemia than in the group with normal SUA levels at every age. The trends in AF prevalence with age were similar between men and women.

### Association between SUA and AF

The association between hyperuricemia and AF was examined by logistic regression analysis (Table 2). Both men and women with hyperuricemia had a higher prevalence of AF than those with normal SUA levels (*P* < 0.001 for men, *P* = 0.010 for women and *P* < 0.001 for total). After adjusting for other cardiovascular and AF risk factors, including age, gender, WC, BMI, systolic and diastolic BP, FBG, TC, TG, smoking, drinking, history of MI, low LVEF, ECG-LVH and familial history of AF, the independent association was significant in men (*P* = 0.003) and in men and women together (*P* = 0.003), but not in women alone (*P* = 0.235). However, *P* > 0.05 was observed for the association of AF with hyperuricemia between men and women. Similar associations were found between SUA as a continuous variable and AF prevalence (Table 3).

**Table 2:** Hyperuricemia and the prevalence of atrial fibrillation

Group	Total	AF	Unadjusted model		Adjusted model^a^	
	(*n*)	*n* (%)	OR (95 % CI)	*P*	OR (95 % CI)	*P*
Men						
Normal SUA levels	4,396	44 (1.0)	1	<0.001	1	0.003
Hyperuricemia	774	20 (2.6)	2.62 (1.54–4.48)		2.55 (1.38–4.71)	
Women						
Normal SUA levels	5,513	60 (1.1)	1	0.010	1	0.235*
Hyperuricemia	655	15 (2.3)	2.13 (1.20–3.77)		1.46 (0.78–2.72)	
Total						
Normal SUA levels	9,909	104 (1.0)	1	<0.001	1	0.003
Hyperuricemia	1,429	35 (2.4)	2.37 (1.61–3.49)		1.94 (1.26–3.00)	

*AF* atrial fibrillation, *CI* confidence interval, *OR* odds ratio, *SUA* serum uric acid

*P = 0.200 for gender difference

^a^Adjusted for age, gender, body mass index, waist circumference, systolic and diastolic blood pressure, fasting blood glucose, total cholesterol and triglyceride levels, smoking, drinking, myocardial infarction, low left ventricular ejection fraction, left ventricular hypertrophy detected by electrocardiography, and family history of AF

**Table 3:** Serum uric acid levels and the prevalence of atrial fibrillation

Group	Unadjusted model		Adjusted model^a^	
	OR (95 % CI)	*P*	OR (95 % CI)	*P*
Men				
SUA, per mg/dl	1.30 (1.13–1.50)	<0.001	1.26 (1.08–1.47)	0.004
Women				
SUA, per mg/dl	1.31 (1.11–1.55)	0.002	1.11 (0.92–1.33)	0.291*
Total				
SUA, per mg/dl	1.25 (1.13–1.38)	<0.001	1.20 (1.06–1.36)	0.003

*AF* atrial fibrillation, *CI* confidence interval, *OR* odds ratio, *SUA* serum uric acid

*P = 0.302 for gender difference

^a^Adjusted for age, gender, body mass index, waist circumference, systolic and diastolic blood pressure, fasting blood glucose, total cholesterol and triglyceride levels, smoking, drinking, myocardial infarction, low left ventricular ejection fraction, left ventricular hypertrophy detected by electrocardiography, and family history of AF

## Discussion

The results of this study demonstrate that men and women with hyperuricemia in a rural Chinese population have a significantly higher prevalence of AF than those with normal SUA levels. The AF prevalence increases with advancing age in both men and women, but the independent association with SUA is only observed in the total population and in men after adjusting for other cardiovascular risk factors. Our finding that hyperuricemia was positively associated with the AF prevalence in the general population was consistent with previous studies [9–13]. However, in our current study, the independent association was only observed in men but not in women, which was inconsistent with previous findings in Japan [9]. A recent meta-analysis that included both cross-sectional and cohort studies, also reported a positive relationship between SUA and the prevalence of AF [20]. Population-based prospective cohort studies showed that baseline SUA was associated with an increased risk of AF in both genders [14, 15]. It is possible that race and lifestyle influenced the results of our study, as Asians and African-Americans have a lower prevalence of AF than other races [21], and our study population consisted of people living exclusively in rural areas, most of whom routinely performed heavy physical labor.

We also tested the difference in the association of SUA with AF between men and women by interaction regression models. However, the effect of gender on the association was not statistically significant with *Ps* > 0.05. This inconsistency may be due to low AF prevalence and the small sample size. Indeed, the positive association between SUA and AF might also exist in women (even though this might not be statistically significant).

In our current study, AF was diagnosed based on a previous history of physician-diagnosed AF and/or evidence of AF using the ECG. This diagnostic approach has been utilized in previous studies [9, 22]. Self-reported AF history was previously used as a method for the diagnosis of AF. However, it is likely that this approach gave rise to an unintentional bias.

The major clinical risk factors for AF are advancing age, male gender, hypertension, diabetes, obesity, heart failure, valve disease, MI, smoking, and alcohol consumption, but biomarkers such as B-type natriuretic peptide [23, 24] and C-reactive protein [25, 26] also correlate with AF. Systemic inflammation indicated by an elevated level of C-reactive protein is associated with the incidence and persistence of AF [26, 27]. SUA regulates some critical proinflammatory pathways [28] and correlates with several inflammatory markers [29]. Inflammation could cause oxidative damage to the atrium that might contribute to electrical remodeling and increase the incidence of AF [30]. SUA may therefore be another useful biomarker for the condition, though the mechanisms by which SUA influences AF risk are unclear.

A limitation of this study is the cross-sectional design and it is not clear whether lowering SUA levels would also decrease the incidence and prevalence of AF. In addition, the number of people who had hyperuricemia in our study was small, and the prevalence of AF in some subgroups was zero or too small to measure for statistical significance. The association between SUA and AF in our study may also have been affected by confounding risk factors for AF (i.e., hypertension, diabetes, and dislipidemia), which also occur with hyperuricemia. However, results from the univariate and multivariate logistic regression analyses indicate that SUA is an independent risk factor for AF. But, further cohort studies need to be conducted to determine whether reducing SUA levels reduce AF incidence and prevalence.

## Conclusions

Men and women in rural China ≥ 35 years of age with hyperuricemia have a significantly higher prevalence of AF than those with normal SUA levels. Hyperuricemia is independently associated with the prevalence of AF after adjusting for various cardiovascular risk factors.
